# Systematics of the Neotropical caddisfly genus *Notidobiella* Schmid (Trichoptera, Sericostomatidae), with the description of 3 new species

**DOI:** 10.3897/zookeys.71.791

**Published:** 2010-12-14

**Authors:** Ralph W. Holzenthal, Roger J. Blahnik

**Affiliations:** Department of Entomology, University of Minnesota, 1980 Folwell Ave. 219 Hodson Hall, St. Paul, Minnesota, 55108, U.S.A.

**Keywords:** caddisfly, Neotropics, transantarctic, new species, biogeography, South America, taxonomy

## Abstract

Three new species of Notidobiella Schmid (Insecta: Trichoptera) are described from South America: Notidobiella amazoniana **sp. n.** (Brazil), Notidobiella brasiliana **sp. n.** (Brazil), and Notidobiella ecuadorensis **sp. n.** (Ecuador). In addition, the 3 previously described species in the genus, Notidobiella chacayana Schmid, Notidobiella inermis Flint, and Notidobiella parallelipipeda Schmid, all endemic to southern Chile, are redescribed and illustrated, including the females of each species for the first time, and a key to males of the species in the genus is provided. The occurrence of Notidobiella in Brazil and Ecuador represents a significant extension of the range of the genus beyond southern Chile where it previously was thought to be endemic. The biogeography of Sericostomatidae and other austral South American Trichoptera is reviewed. The presence of the family in South America may not be part of a “transantarctic” exchange, but instead may represent an earlier occurence in the region. The distribution of Notidobiella in tropical South America likely represents recent dispersal from southern South America to the north.

## Introduction

The caddisfly family Sericostomatidae occurs in all biogeographic regions, except the Australasian, but its species diversity is very unevenly distributed across these regions (Morse 2010). Nineteen genera and 100 species have been described world wide, with half of the species occurring in the western Palearctic (50 species in 5 genera: Cerasma McLachlan, Notidobia Stephens, Oecismus McLachlan, Schizopelex McLachlan, Sericostoma Latreille) (Holzenthal et al. 2007b). South Africa harbors 12 species in 5 endemic genera (Aclosma Morse, Aselas Barnard, Cheimacheramus Barnard, Petroplax Barnard, Rhoizema Barnard), but no species are found in tropical Africa. The genus Agarodes Banks contains 12 species confined largely to the southeastern United States, where a second monotypic genus, Fattigia Ross, also occurs. There is a single species known from India, Asahaya asambaddha Schmid, and the 6 species in the genus Gumaga Tsuda occur in Mexico (Baja California) and the western United States (3 species) and in southern and eastern Asia (3 species). In the Neotropics, the family is represented in the Chilean subregion by 4 genera, Chiloecia Navás (1 species, *nomen dubium*), Myotrichia Schmid (1), Notidobiella Schmid (3), and Parasericostoma Schmid (10) (Flint et al. 1999b). Until now, only a single sericostomatid species, Grumicha grumicha (Vallot), was known from the Brazilian subregion (Flint et al. 1999a).

In this paper, we describe 3 new species of Notidobiella, 1 from the Amazon basin, Brazil, 1 from southeastern Brazil, and 1 from Ecuador, thus extending the range of this genus well beyond its Chilean representation. In addition, we provide illustrations and diagnoses of males and females (the latter for the first time) of the 3 previously described species of Notidobiella, Notidobiella chacayana Schmid, Notidobiella inermis Flint, and Notidobiella parallelipipeda Schmid, and a key to males of species in the genus. The Neotropical species of Sericostomatidae, including those in the genus Notidobiella, appear to be members of a southern Gondwana fauna (de Moor and Ivanov 2008).

## Material and methods

Techniques and procedures used in the preparation and examination of specimens are those outlined by Blahnik and Holzenthal (2004) and Blahnik et al. (2007). Terminology for genitalia and wing venation follows that presented by Holzenthal et al. (2007b) and morphological structures are labeled in Figs 3–5. The species are presented in alphabetical order, except for the type species of the genus, Notidobiella parallelipipeda, which is presented first, after the generic diagnosis. Material examined and types are deposited in the collections of the University of Minnesota Insect Collection, St. Paul, Minnesota, USA (UMSP), the Museu de Zoologia, Universidade de São Paulo, São Paulo, Brazil (MZUSP), the Instituto Nacional de Pesquisas da Amazonia, Manaus, Brazil (INPA), and the National Museum of Natural History, Smithsonian Institution, Washington, DC, USA (NMNH). UMSP barcode accession label numbers for holotypes are included in the list of material examined, but not for paratypes.

## Systematics

### Genus Notidobiella

Schmid

Notidobiella Schmid 1955: 152 [Type species: Notidobiella parallelipipedaSchmid 1955, original designation].

#### Diagnosis:

**Figures 1–3.**
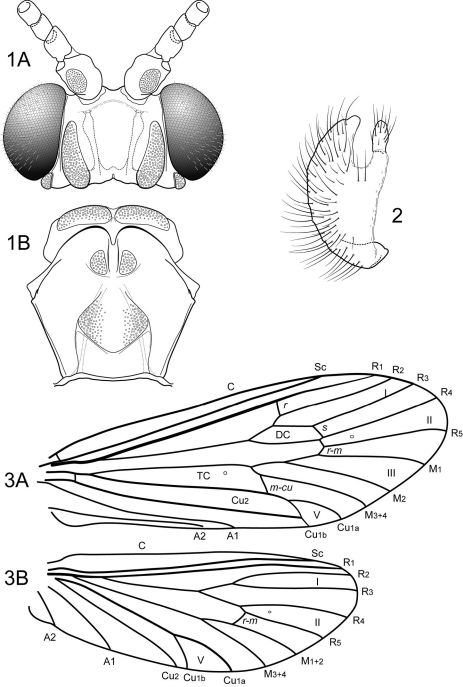
Notidobiella parallelipipeda Schmid. **1** Male head and thorax **A** head, dorsal **B** pro- and mesonota, dorsal. **2.** Notidobiella parallelipipeda Schmid. Maxillary palp, male, frontal view. **3.** Notidobiella parallelipipeda Schmid. Male wings **A** forewing **B** hind wing. Abbreviations: DC = discoidal cell, TC = thyridial cell.

**Figures 4–5.**
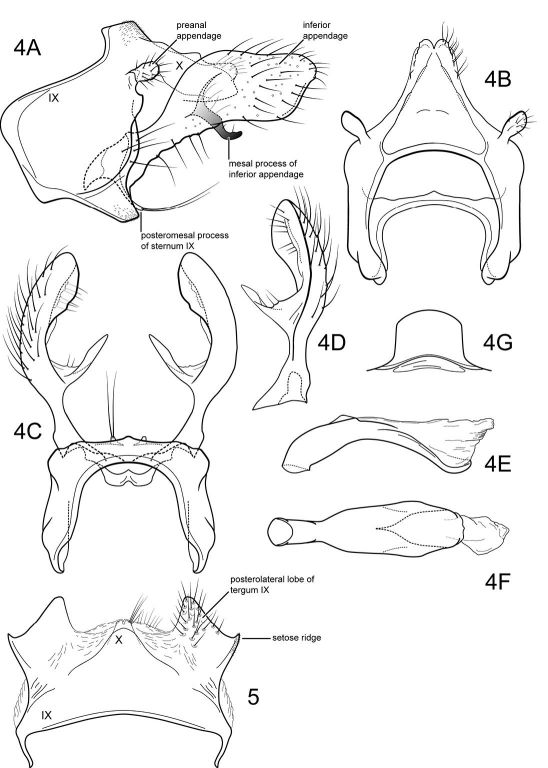
Notidobiella parallelipipeda Schmid. **4** Male genitalia **A** segments IX, X, inferior appendages, lateral **B** segments IX, X, dorsal **C** segment IX, inferior appendages ventral **D** inferior appendage, dorsal **E** phallus, lateral **F** phallus, ventral **G** sternum VII posteromesal process, ventral. **5.** Notidobiella parallelipipeda Schmid. Female genitalia, segments IX, X, dorsal.

(modified from Schmid 1955; characters pertain to the male of the type species, except where noted): Head short, but broad, with large, projecting eyes, with conspicuous setae between ommatidea (Fig. 1); in most species interocular distance equal to or slightly less than diameter of eye, when viewed frontally (eyes of females smaller, interocular distance about 2× diameter of eye). According to Schmid (1955) bordering the eyes medially is an elongate, convex cephalic tubercle, but this structure was not evident in the material of the type species examined by us. Head dorsally with prominent, elongate occipital and retroccipital warts; ocellar, antennal, frontal, anterolateral, and hypomedial setal warts absent (terminology of Ivanov 1990) (although females with small anterolateral setal wart). Antennal scape much shorter than head and about as wide as long, with oval setal warts on both its dorsal and ventral surfaces; scapes almost touch medially. Maxillary palp very short, composed of enlarged, heavily setose basal article held against the face and sclerotized on its lateral side only, medial side membranous, with small, oval, sclerotized, setose apical article (Fig. 2) (female maxillary palp 5-segmented, unmodified). Labial palp 3-segmented, unmodified. Pronotum with pair of elongate setal warts (Fig. 1). Mesoscutum with very deep median fissure and pair of small, oval scutal warts; mesoscutellum with pair of large scutellar warts (Fig. 1). Each leg with pair of apical spurs (tibial spur formula 2-2-2). Wings are broad, forewing longer and broader than hind wing; wing venation uniform among the species. Forewing (Fig. 3A) with forks I, II, III, and V present; cross vein *r* between R1 and R2; discoidal cell present and short, forks II and III sessile; crossveins *r-m* and *m-cu* present (in Notidobiella amazoniana sp. n., and Notidobiella ecuadorensis sp. n., crossveins *r* and *s* absent or very weak and difficult to discern in the material examined; the absence of *s* leaves the discoidal cell open); Cu2 not attaining wing margin, merged apically with Cu1b; A1 and A2 each attaining wing margin, A3 absent. Hind wing (Fig. 3B) with discoidal cell open; forks I, II, V present; fork I petiolate; fork II sessile; M with single branch; fork V short. Male genitalia (Figs 4, 8): Sternum VII with posteromesal process. Segment IX with short or long ventral setose lobes or only single setose process (Notidobiella amazoniana sp. n.). Preanal appendages short, ovate, setose. Tergum X simple, triangular to subquadrate in shape. Inferior appendage narrow basally and broadly spatulate apically, with short to long mesal process on ventromesal margin (in Notidobiella amazoniana sp. n., inferior appendage uniformly narrow throughout its length and without mesal process on the ventromesal margin). Phallus simple, elongate, tubular, with prominent endophallic membranes. Female genitalia (Figs 5, 9): Tergum IX heavily setose, posterolateral lobe, with lateral, microsetose, elevated ridge (all species except Notidobiella brasiliana sp. n.). Internal vaginal sclerites complex with no discernable differences among the species. Bursa copulatix subspherical and semisclerotized; pair of small, oval sclerites lying in membranes above vaginal sclerites (position variable).

Flint (1967) described the immature stages of Notidobiella chacayana. Larvae construct slightly tapered and curved cases of small mineral fragments embedded in silk and occur on the bottoms of small streams. The 3 previously known species, Notidobiella chacayana Schmid, 1957, Notidobiella inermis Flint, 1983, and Notidobiella parallelipipeda Schmid, 1955, are endemic to southern Chile.

### Notidobiella parallelipipeda

Schmid

Figs 1 5

Notidobiella parallelipipeda Schmid 1955: 152 [Type locality: Chile, Ñuble, Recinto; NMNH; male]. Flint 1974: 91 [distribution].

#### Description.

Of the species in the genus with broad, spatulate inferior appendages (all species except Notidobiella amazoniana), the type species is the most distinctive based on the parallel-sided inferior appendages with their prominent, mesally directed, mesal processes.

##### Adult.

Forewing length 7.8–8.0 mm male (n=2); 8.8–9.0 mm female (n=2). Color light brown, palps and legs stramineous; forewings light brown, with scattered golden setae. Sternum VII of male with broad, fingernail-like, posteromesal process.

##### Male genitalia

(Fig. 4). Segment IX with anterior margin broadly produced midlaterally; tergum IX narrow; sternum IX with pair of very short, posteromesal processes, bearing long apical setae. Tergum X simple, subquadrate in lateral view, with slight apicolateral elevation, with slight dorsomesal excavation, setose apically. Preanal appendage short, ovate, setose. Inferior appendage prominent, heavily setose, spatulate, dorsal and ventral edges parallel, narrow basally, with prominent, elongate mesal process on mesal margin; strongly directed mesally in ventral view; apex exposed in lateral view. Phallic apparatus simple, tubular, slightly curved from base to apex; endophalic membranes prominent, but simple; phallotremal sclerite not apparent.

##### Female genitalia

(Fig. 5). Tergum VIII quadrate; pleural membranes extensive, highly folded; sternum VIII broad, anterior margin with apodemal ridge, extending dorsolaterally; posterolateral corners rounded, heavily setose, especially posteriorly. Tergum IX with heavily setose, posterolateral lobes, rounded in lateral view, triangular in dorsal view; with lateral, microsetose, elevated ridge; sternum IX highly membranous, the membranes with parallel pleats or folds; tergum IX semimembranous dorsally. Tergum X with short setose projection. Internal vaginal sclerites complex (no discernable differences among the species); bursa copulatix subspherical, semisclerotized; pair of small, oval sclerites lying in membranes above vaginal sclerites (position variable).

#### Material Examined:

**CHILE:** Ñuble, Recinto, 4–6.iii.1968, Flint and Peña, 1 male, 1 female (pinned) (NMNH); Linares, El Castillo, Malcho, E Parral, 750 m, 8–10.i.1988, L.E. Peña, 1 male, 1 female (pinned) (NMNH).

### Notidobiella amazoniana

Holzenthal & Blahnik sp. n.

urn:lsid:zoobank.org:act:6203A2A2-CB58-418F-A731-8C31A2243E7D

Figs 6 7 

#### Description.

This is the smallest species in the genus. Its wings are short and broad with venation typical for the genus except forewing crossveins *r* and *s* are absent, leaving the discoidal cell open (Fig. 7). Its genitalia are the most atypical in the genus in that the inferior appendages are not broadly spatulate, but sinuous in shape and uniform in width. Tergum IX bears a short triangular, posteromesal process, rather than short, paired processes, as found in the other species. Otherwise, the genitalia are typical for the genus.

##### Adult.

Forewing length 4.5–5.0 mm male (n=8). Color faded, overall pale stramineous (specimens in alcohol); forewings colorless, almost transparent, denuded. Sternum VII of male with broad, fingernail-like, posteromesal process.

##### Male genitalia

**Figure 6.**
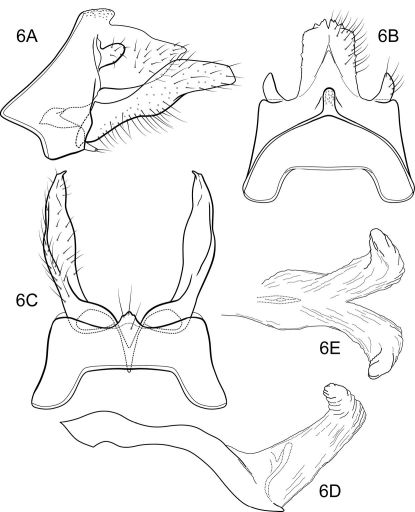
Notidobiella amazoniana, sp. n. Male genitalia **A** segments IX, X, inferior appendages, lateral **B** segments IX, X, dorsal **C** segment IX, inferior appendages ventral **D** phallus, lateral **E** apex of phallus, dorsal.

(Fig. 6). Segment IX with anterior margin acutely produced ventrolaterally; tergum IX narrow, ridge-like; sternum IX with short, triangular, posteromesal process, bearing apical setae. Tergum X simple, subquadrate in lateral view, with slight dorsomesal excavation, setose. Preanal appendage short, ovate, setose. Inferior appendage prominent, heavily setose, elongate, narrow throughout length, without mesal process on ventromesal margin; in ventral view, apex acute, slightly incurved. Phallic apparatus simple, tubular, slightly curved from base to apex; endophalic membranes prominent, with paired apical membranous lobes; elongate, lightly sclerotized band internally (perhaps the phallotremal sclerite).

##### Female.

Unknown.

**Figure 7.**
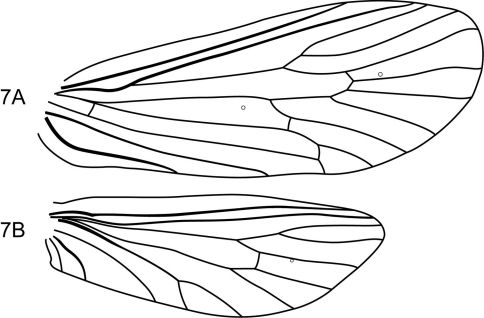
Notidobiella amazoniana, sp. n. Wings **A** forewing **B** hind wing.

#### Holotype male:

**BRAZIL: Amazonas:** AM 010, km 246, 15–16.vii.1979, J. Arias (alcohol) (UMSP000131226) (INPA).

#### Paratypes:

**BRAZIL: Amazonas:** same data as holotype, 3 males (alcohol) (UMSP), 4 males (alcohol) (NMNH).

#### Etymology:

Named for the state and region where the specimens were collected, which represents a significant northern extension of the range of the genus.

### Notidobiella brasiliana

Holzenthal & Blahnik sp. n.

urn:lsid:zoobank.org:act:3365A48E-060D-4925-AC4B-B8AD7E74944F

Figs 8 [Fig F6] 10 

#### Description.

This new species is most similar to Notidobiella chacayana in the overall shape and structure of the inferior appendages. Both species possess an elongate mesal process on the ventromesal margin of the inferior appendage. In Notidobiella ecuadorensis sp. n., the ventromesal process is also present, but is shorter and broader in ventral view; in the other 2 Chilean species, Notidobiella inermis and Notidobiella parallelipipeda, the ventromesal processes are either very reduced (Notidobiella inermis) or long (Notidobiella parallelipipeda), but not nearly as long as in Notidobiella brasiliana sp. n. Setting Notidobiella brasiliana sp. n., apart from all of its congeners is the pair of elongate posteromesal processes on sternum IX; in all other species these processes are much shorter and broader. Furthermore, forewing crossveins *r* and *s* are absent, leaving the discoidal cell open (Fig. 10).

##### Adult.

Forewing length 7.0 mm male (n=1); 7.9–8.2 mm female (n=4). Color medium to dark brown, palps and legs light brown; forewings dark brown with scattered golden hairs, pale golden spot on anal margin at about midlength. Sternum VII of male with broad, fingernail-like, posteromesal process.

##### Male genitalia

**Figure 8.**
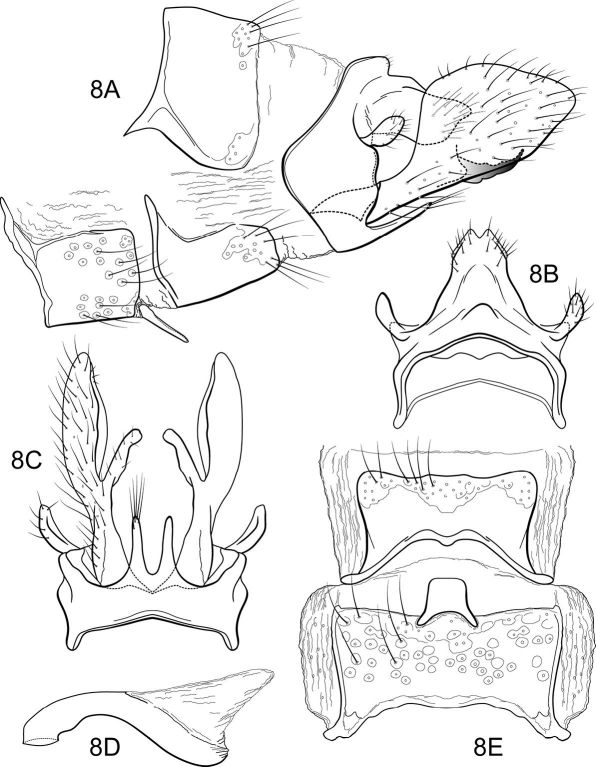
Notidobiella brasiliana, sp. n. Male genitalia **A** segments VII-X, inferior appendages, lateral **B** segments IX, X, dorsal **C** segment IX, inferior appendages ventral **D** phallus, lateral **E** sterna VII, VIII, ventral.

(Fig. 8). Segment IX with anterior margin broadly produced mesolaterally; tergum IX narrow, elevated, mound-like; sternum IX with pair of prominent, elongate, posteromesal processes, bearing long apical setae. Tergum X simple, triangular in lateral view, with slight dorsomesal excavation, setose. Preanal appendage short, ovate, setose. Inferior appendage prominent, heavily setose, broadly spatulate, narrow basally, with elongate mesal process on ventromesal margin. Phallic apparatus simple, tubular, slightly curved from base to apex; endophalic membranes prominent, but simple; phallotremal sclerite not apparent.

##### Female genitalia

**Figure 9. F6:**
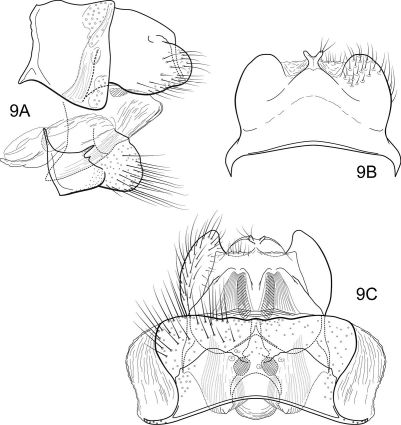
Notidobiella brasiliana, sp. n. Female genitalia **A** segments VIII-X, lateral **B** segments IX, X, dorsal **C** segments VIII-X, ventral.

(Fig. 9). Tergum VIII quadrate; pleural membranes extensive, highly folded; sternum VIII broad, anterior margin with apodemal ridge, extending dorsolaterally; posterolateral corners rounded, heavily setose, especially posteriorly. Tergum IX with heavily setose, posterolateral lobes, rounded in dorsal and lateral views; without lateral ridge; sternum IX highly membranous, the membranes with parallel pleats or folds; tergum IX semimembranous dorsally. Tergum X with short, bifurcate, setose projection. Internal vaginal sclerites complex (no discernable differences among the species); bursa copulatix subspherical, semisclerotized; pair of small, oval sclerites lying in membranes above vaginal sclerites (position variable).

**Figure 10.**
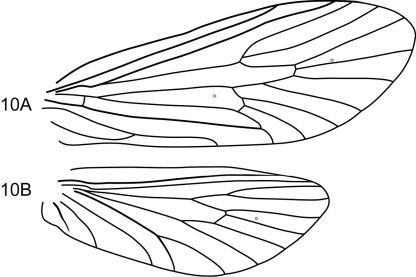
Notidobiella brasiliana, sp. n. Wings **A** forewing **B** hind wing.

#### Holotype male:

**BRAZIL: São Paulo:** Parque Estadual de Campos do Jordão, 1st order trib. to Rio Galharada, 22°41.662'S, 45°27.783'W, el 1530 m, 14–16.ix.2002, Blahnik, Prather, Huamantinco (pinned) (UMSP000086351) (MZUSP).

#### Paratypes:

**BRAZIL: São Paulo:** Parque Estadual de Campos do Jordão, Rio Galharada, 22°41.662'S, 45°27.783'W, el 1530 m, 13–15.ix.2002, Blahnik, Prather, Melo, Huamantinco, 2 females (alcohol) (MZUSP); same data as holotype, 2 females (pinned) (UMSP).

#### Etymology:

Named for Brazil, the country of the type specimens, which represents a significant northeastward extension of the range of the genus.

### Notidobiella chacayana

Schmid

Figs 11–12

Notidobiella chacayana Schmid 1957: 392 [Type locality: Chile, Maule, Chacay; NMNH; male]. Flint 1967: 63 [larva, pupa]; 1974: 91 [distribution].

#### Description.

This Chilean species is most similar to Notidobiella brasiliana because of the similarly shaped inferior appendages, with their similar elongate mesal processes. It differs from that species in the much shorter posteromesal processes of sternum IX.

##### Adult.

Forewing length 6.8–7.5 mm male (n=3); 7.2–9.0 mm female (n=3). Color brown, palps and legs stramineous; forewings brown, with scattered golden setae. Sternum VII of male with broad, fingernail-like, posteromesal process.

##### Male genitalia

**Figures 11–12.**
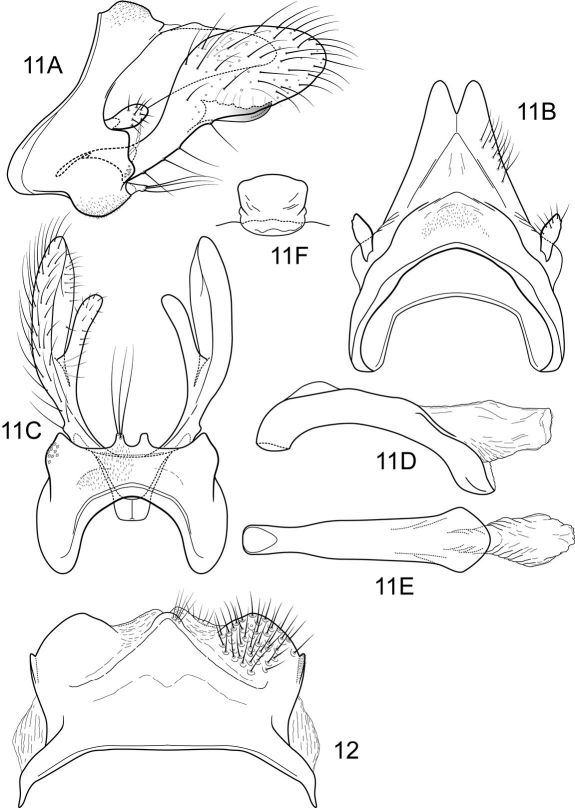
Notidobiella chacayana Schmid. **11** Male genitalia **A** segments IX, X, inferior appendages, lateral **B** segments IX, X, dorsal **C** segment IX, inferior appendages ventral **D** phallus, lateral **E** phallus, ventral. **12.** Notidobiella chacayana Schmid. Female genitalia, segments IX, X, dorsal.

(Fig. 11). Segment IX with anterior margin produced ventrolaterally; tergum IX slightly elevated, mound-like; sternum IX with pair of short, posteromesal processes, bearing long apical setae. Tergum X simple, triangular in lateral view, with dorsomesal excavation, setose. Preanal appendage short, ovate, setose. Inferior appendage prominent, heavily setose, broadly spatulate, narrow basally, with elongate mesal process on ventromesal margin. Phallic apparatus simple, tubular, curved from base to apex; endophalic membranes prominent, but simple; phallotremal sclerite not apparent.

##### Female genitalia

(Fig. 12). Tergum VIII quadrate; pleural membranes extensive, highly folded; sternum VIII broad, anterior margin with apodemal ridge, extending dorsolaterally; posterolateral corners rounded, heavily setose, especially posteriorly. Tergum IX with heavily setose, posterolateral lobes, rounded to subtriangular in dorsal view; with lateral, microsetose, elevated ridge; sternum IX highly membranous, the membranes with parallel pleats or folds; dorsally tergum IX semimembranous. Tergum X with short setose projection. Internal vaginal sclerites complex (no discernable differences among the species); bursa copulatix subspherical, semisclerotized; pair of small, oval sclerites lying in membranes above vaginal sclerites (position variable).

#### Material Examined:

**CHILE**: Cauquenes, Tregualeme, 35°56'S, 72°43'W, 11–12.xii.1993, C. and O. Flint Jr., 1 male, 1 female (pinned) (NMNH); X Región de los Lagos, Isla de Chiloé, Río Verde, 1.9 km W Puntra, 42°07.078'S, 73°50.364'W, el. 40 m, 3.ii.2005, Holzenthal, Blahnik, Chamorro, 2 males, 2 females (pinned) (UMSP); XIV Región de los Ríos, Monumento Nacional Alerce Costero, unnamed trib., trail to Alerce Milenario, 40°11.874'S, 73°26.217'W, el. 895 m, 5.ii.2008, Holzenthal, Pauls, Mendez, 1 male (pinned) (UMSP).

### Notidobiella ecuadorensis

Holzenthal & Blahnik sp. n.

urn:lsid:zoobank.org:act:20CB7DC2-73DF-4D4D-9593-0A87C589BABB

Figs 13 14 

#### Description.

The combination of broadly spatulate inferior appendage, thumb-like mesal process on the ventromesal margin of the inferior appendage, and short posteromesal processes on sternum IX separate this species from its congeners. The wing venation (Fig. 14) is similar to that of the type species.

##### Adult.

Forewing length 6.2 mm (n=1). Color faded, overall yellowish-brown (specimen in alcohol); forewings stramineous, denuded. Sternum VII of male with broad, fingernail-like, posteromesal process.

##### Male genitalia

**Figure 13.**
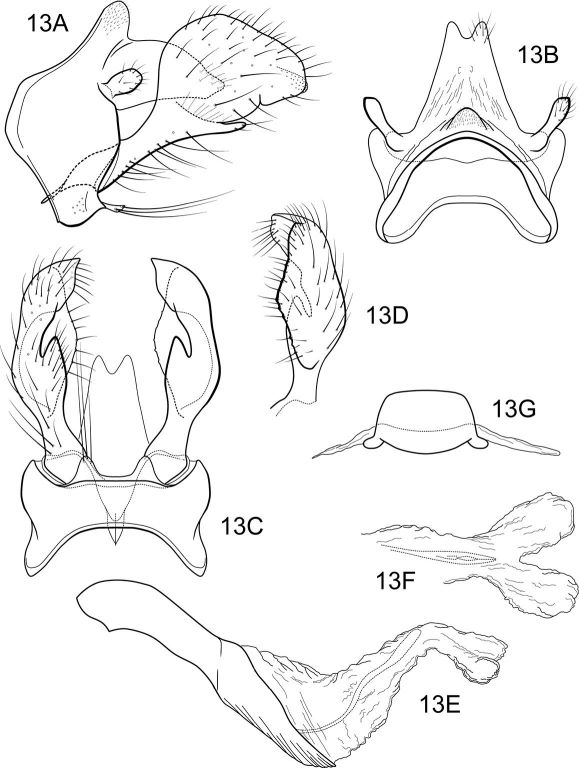
Notidobiella ecuadorensis, sp. n. Male genitalia **A** segments IX, X, inferior appendages, lateral **B** segments IX, X, dorsal **C** segment IX, inferior appendages ventral **D** inferior appendage, dorsal **E** phallus, lateral **F** phallus apex, dorsal **G** sternum VII posteromesal process, ventral.

(Fig. 13). Segment IX with anterior margin broadly produced midlaterally; tergum IX narrow, elevated, mound-like; sternum IX with pair of short, triangular, posteromesal processes, bearing very long apical setae. Tergum X simple, triangular in lateral view, with slight dorsomesal excavation, setose apically. Preanal appendage short, ovate, setose. Inferior appendage prominent, heavily setose, very broadly spatulate, narrow basally, with short, thumb-like mesal process on ventromesal margin. Phallic apparatus simple, tubular, relatively straight from base to apex; endophalic membranes prominent, with paired apical membranous lobes; elongate, lightly sclerotized band internally (perhaps the phallotremal sclerite).

##### Female:

Unknown.

**Figure 14.**
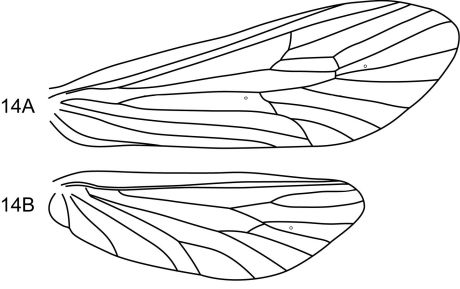
Notidobiella ecuadorensis, sp. n. Wings **A** forewing **B** hind wing.

#### Holotype:

male, **ECUADOR:** **Pastaza**: Puyo, 1–7.ii.1976, Spangler et al. (alcohol) (UMSP000208470) (NMNH).

#### Etymology:

Named for Ecuador, the country of the holotype, which represents a significant northern extension of the range of the genus.

### Notidobiella inermis

Flint

Figs 15–16

Notidobiella inermis Flint 1983:90 [Type locality: Chile, Pcia Cautín, near Pucón; NMNH; male].

#### Description.

Notidobiella inermis shares with Notidobiella ecuadorensis broadly spatulate inferior appendages with short, thumb-like mesal processes, but differs in details of the shape of the inferior appendages, as illustrated, and in the possession of a narrow posteromesal process on sternum VII.

##### Adult.

Forewing length 6.0–6.5 mm male (n=2); 8.0 mm female (n=2). Color brown, palps and legs stramineous; forewings brown, with scattered golden setae. Sternum VII of male with narrow, fingernail-like, posteromesal process.

##### Male genitalia

**Figures 15–16.**
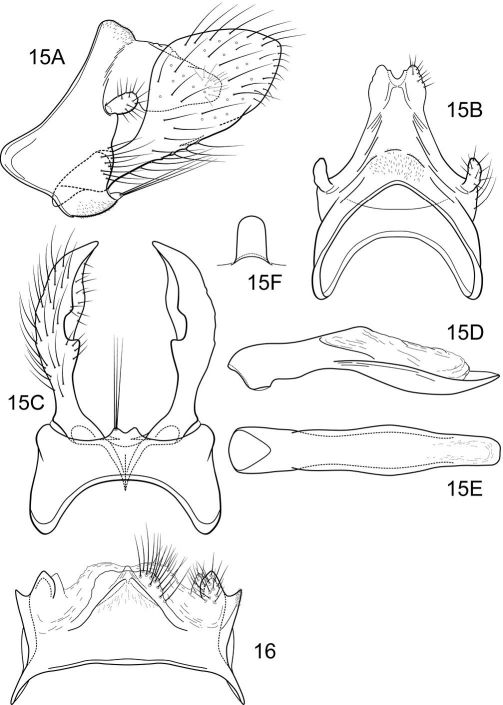
Notidobiella inermis Flint. **15** Male genitalia **A** segments IX, X, inferior appendages, lateral **B** segments IX, X, dorsal **C** segment IX, inferior appendages ventral **D** phallus, lateral **E** phallus, ventral **F** sternum VII posteromesal process, ventral. **16.** Notidobiella chacayana Schmid. Female genitalia, segments IX, X, dorsal.

(Fig. 15). Segment IX with anterior margin produced ventrolaterally; tergum IX narrow; sternum IX with pair of short, posteromesal processes, bearing long apical setae. Tergum X simple, subquadrate in lateral view, with slight dorsomesal excavation, setose. Preanal appendage short, ovate, setose. Inferior appendage prominent, heavily setose, very broadly spatulate, narrow basally, with short, thumb-like mesal process on ventromesal margin. Phallic apparatus simple, tubular, relatively straight from base to apex; endophalic membranes prominent, but simple; phallotremal sclerite not apparent.

##### Female genitalia

(Fig. 16). Tergum VIII quadrate; pleural membranes extensive, highly folded; sternum VIII broad, anterior margin with apodemal ridge, extending dorsolaterally; posterolateral corners rounded, heavily setose, especially posteriorly. Tergum IX with heavily setose, posterolateral lobes, subovate, small, bilobed in dorsal and lateral views; with lateral, microsetose, elevated ridge; sternum IX highly membranous, the membranes with parallel pleats or folds; dorsally tergum IX with sclerotized ridge. Tergum X with broad heavily setose projection. Internal vaginal sclerites complex, no discernable differences among the species; bursa copulatix subspherical, semisclerotized; pair of small, oval sclerites lying in membranes above vaginal sclerites (position variable).

#### Material Examined:

**CHILE:** Llanquihue, Salto Chamiza, Correntosa, 100 m, 19.i.1987, C.M. and O.S. Flint, Jr., 1 male, 2 females (pinned) (NMNH); Llanquihue, El Chinque, N Correntosa (S Volcán Calbuco), 300 m, 20–25.i.1980, 1 male paratype (pinned) (NMNH).

## Key to males of Neotropical Notidobiella

**Table:** 

1	Inferior appendage narrow basally, broadly spatulate apically, with mesal process on ventromesal margin (Figs 4A, 8A); abdominal sternum IX with pair of posteromesal processes (Figs 4C, 8C); forewing length 6–8 mm	2
–	Inferior appendage elongate, narrow throughout length, without mesal process on ventromesal margin (Figs 6A, C); abdominal sternum IX with single, short, triangular, posteromesal process (Fig. 6C); forewing length 4.5–5 mm	Notidobiella amazoniana sp. n.
2(1)	Inferior appendage mesal process elongate (Figs 4C, 8C)	3
–	Inferior appendage mesal process short, thumb-like (Figs 13C, 15C)	5
3(2)	Posteromesal processes of abdominal sternum IX short (Figs 6C, 13C); distribution: Chilean subregion (Chile)	4
–	Posteromesal processes of abdominal sternum IX elongate (Fig. 8C); distribution: Brazilian subregion (southeastern Brazil)	Notidobiella brasiliana sp. n.
4(3)	Spatulate apex of inferior appendage broadly ovate (Fig. 11A)	Notidobiella chacayana Schmid
–	Spatulate apex of inferior appendage parallel sided (Fig. 4A)	Notidobiella parallelipipeda Schmid
5(2)	Abdominal tergum IX highly elevated, mound-like (Fig. 13A); ventromesal process of abdominal sternum VII broad (Fig. 13G); distribution: Brazilian subregion (Ecuador)	Notidobiella ecuadorensis sp. n.
–	Abdominal tergum IX not elevated (Fig. 15A); ventromesal process of abdominal sternum VII narrow (Fig. 15F); distribution: Chilean subregion (Chile)	Notidobiella inermis Flint

## Biogeographic considerations

As defined most recently by de Moor and Ivanov (2008), the Trichoptera fauna of southern Chile and adjacent patagonian Argentina exhibits a strong biogeographical affinity to Australia, New Zealand, and other southern Pacific islands (e.g., New Caledonia). This “Temperate Gondwana” (de Moor and Ivanov 2008) or “transantarctic” pattern (e.g., Brundin 1966) also includes the temperate, southernmost part of Africa and Madagascar. In the Neotropical Trichoptera, the affinity to the southern African fauna is very weak, perhaps exhibited only at the family level within Sericostomatoidea (de Moor and Ivanov 2008) and potentially among genera within Sericostomatidae (although relationships among genera within this family are yet to be inferred). On the other hand, the biogeographical affinity between southern South America and Australasia is strong. Within South America, nearly all species in Chile and adjacent Argentina  are endemic, prompting Flint (1976) to divide the Neotropics into 2 distinct subregions, the Chilean (southern Chile/Argentina) and the Brazilian (the rest of the Neotropics as defined by Wallace 1876); these regions are equivalent to the “Patagonian” and “Neotropical” (*sensu stricto*) Trichoptera regions of de Moor and Ivanov (2008).

Four Trichoptera families are representative of a temperate Gondwanan pattern: Helicophidae, Kokiriidae, Philorheithridae, and Tasimiidae, each family with genera endemic to Australia/New Zealand/New Caledonia, southern South America, or Madagascar (no genera are shared) (Neboiss 1986, Flint et al. 1999b, Holzenthal et al. 2007b, Weaver et al. 2008) (Table 1). Sukatcheva and Jarzembowski (2001) questionably placed a fossil (fragment of a forewing) from the early Cretaceous of southern England in the Helicophidae.

**Table 1.** Genera (number of included species) in the families Helicophidae, Kokiriidae, Philorheithridae, and Tasimiidae and their regional distributions, including references to recent works inferring or discussing phylogenetic relationships among genera.

Family, genus (# species)	Distribution
Helicophidae (Flint 1992, 2002, Henderson and Ward 2007, Johanson 2003a, Johanson and Keijsner 2008, Johanson and Ward 2002, Neboiss 2002)	
Alloecella Banks (3)	SE Australia, Tasmania
Alloecentrella Wise (4)	New Zealand
Alloecentrellodes Flint (2)	Chile
Austrocentrus Schmid (3)	Chile, Argentina
Eosericostoma Schmid (2)	Chile, Argentina
Helicopha Mosely (21)	Australia, Tasmania, New Caledonia
Heloccabus Neboiss (1)	E Australia
Microthremma Schmid (8)	Chile
Pseudosericostoma Schmid (1)	Chile
Zelolessica McFarlane (2)	New Zealand (incl. Stewart Island)
Kokiriidae (Johanson 2003b)	
Kokiria McFarlane (1)	New Zealand
Mecynostomella Kimmins (7)	New Caledonia
Pangulia Navás (2)	Chile
Tanjistomella Neboiss (1)	SE Australia
Taskiria Neboiss(3)	SE Australia, Tasmania
Taskiropsyche Neboiss (1)	Tasmania
Philorheithridae (Henderson and Ward 2006, Weaver et al. 2008)	
Afrorheithrus Weaver, Gibon, and Chvojka (3)	Madagascar
Aphilorheithrus Mosely (4)	SE Australia, Tasmania
Austrheithrus Mosely (3)	SE Australia, Tasmania
Kosrheithrus Mosely (3)	SE, SW Australia, Tasmania
Mystacopsyche Schmid (2)	Chile, Argentina
Philorheithrus Hare (6)	New Zealand
Psilopsyche Ulmer (3)	Chile, Argentina
Ramiheithrus Neboiss (2)	SE Australia, Tasmania
Tasmanthrus Mosely (3)	Tasmania
Tasimiidae (no phylogenetic assessment available)	
Charadropsyche Flint (1)	Chile
Tasiagma Neboiss (2)	SE Australia, Tasmania, Lord Howe Island
Tasimia Mosely (5)	SE Australia, Tasmania
Trichovespula Schmid (1)	Chile

While not wholly endemic to the region, other caddisfly families contain a diverse temperate Gondwana fauna including, most notably, Hydrobiosidae (reviewed by Schmid 1989, Ward et al. 2004), Limnephilidae: Dicosmoecinae (Wiggins 2002) and Leptoceridae: Triplectidinae (Holzenthal 1986a, b, Morse and Holzenthal 1987, de Moor 1997, Holzenthal and Pes 2004, Calor et al. 2006, Calor and Holzenthal 2008, Malm and Johanson 2008). Other extant caddisfly taxa conforming to a temperate Gondwanan pattern between the Neotropics (*sensu* Wallace) and Australasia include closely related or purported sister genera in otherwise more widely distributed or cosmopolitan families, including genera in Ecnomidae (Flint 1973, Li and Morse 1997, Cartwright 2009, Johanson and Espeland 2009), Hydropsychidae: Smicrideinae, Macronematinae (Neboiss 1984, Schefter 1996, Geraci et al. 2005), Philopotamidae (Blahnik 2005), and possibly Stenopsychidae. Other Trichoptera taxa endemic to the Patagonian region (as defined by de Moor and Ivanov 2008), but that apparently either do not pertain to a transantarctic pattern or have unknown biogeographic affinities include genera in Anomalopsychidae (Holzenthal and Flint 1995, Holzenthal and Robertson 2006), Glossosomatidae (Robertson and Holzenthal 2005), Leptoceridae: Leptocerinae (Holzenthal 1986c), and Hydroptilidae (Harris and Armitage 1997, Harris and Flint 1993).

The family Sericostomatidae contains temperate Gondwanan components, including 5 endemic South African/Malagasy genera (Aclosma, Aselas, Cheimacheramus, Petroplax, Rhoizema) and 4 endemic South American genera (Grumicha, Myotrichia, Notidobiella, Parasericostoma, excluding Chiloecia, *nomen dubium*). As indicated above, the family includes other genera endemic to the Nearctic and West Palearctic regions. Sericostomatids are absent from the Australasian region (all Australasian species previously assigned to Sericostomatidae have been transferred to other families, see Holzenthal et al. 2007b for a historical review). Phylogenetic relationships among families and genera of Sericostomatoidea are largely unresolved (Holzenthal et al. 2007a) making it impossible to construct an area cladogram to test for congruence with the prevailing hypotheses of the geological sequence of the breakup of Pangea or the subsequent breakup of southern Gondwana (Sanmartín and Ronquist 2004, fig. 1).

The presence of Helicophidae and Hydrobiosidae in Eocene Baltic amber (Botosaneanu and Wichard 1983) and of fossil Plectrotarsidae (extant taxa endemic to Australasia) and a putative helicophid from late Cretaceous deposits in England (Sukatsheva and Jarzembowski 2001) suggests, as hypothesized by de Moor and Ivanov (2008), that certain southern temperate Trichoptera may be relicts of a more widespread fauna which included now extinct (but still extant in Sericostomatidae) north temperate elements. Two species of Triplectides in Baltic amber (Ulmer 1912) suggests the same scenario for this southern Gondwanan genus. In addition, the putative triplectidine larva from South Africa (de Moor 1997) and the recent discovery of Philorheithridae in Madagascar (Weaver et al. 2008) suggest at least a Gondwanan origin for these taxa (category 3 of Amorin et al. 2009) and, by inference, other endemic austral Trichoptera.

Evidence suggests that the contemporary distribution of the Patagonian and Australasian temperate Gondwanan Trichoptera fauna reflects a past dispersal corridor between Australia and southern South America via Antarctica (Sanmartín and Ronquist 2004). This “transantarctic exchange” pattern also has been demonstrated for other insects, including aquatic taxa (Cranston and Edward 1999, Amorin et al. 2009, Daugeron et al. 2009). However, it may be that the current distribution of other southern Gondwana caddisflies, such as Sericostomatidae, reflects an older dispersal prior to the breakup of southern Gondwana, now represented by relict Southern Hemisphere distributions. The southeast Brazilian sericostomatid Grumicha grumicha might represent evidence to support the relict hypothesis.

The now widespread occurrence of Notidobiella in temperate southern Chile and tropical South America (Ecuador, southeast Brazil, Amazonian Brazil) suggests a more recent dispersal of the genus to northern tropical South America from Patagonia and its subsequent diversification. The data from insects analyzed by Sanmartín and Ronquist (2004, table 4) found a significantly higher frequency of dispersal from southern South America to northern South America than from the other direction. This may be true for other caddisflies with both Patagonian and Neotropical (*sensu* de Moor and Ivanov) distributions, including Antarctoecia (Huamantinco and Nessimian 2003), Atopsyche and Cailoma (Hydrobiosidae) (Ross and King 1952, Flint 1974, Schmid 1989 [although Atopsyche is absent from Patagonia, its putative sister genus is Patagonian]), Contulma (Anomalopsychidae) (Holzenthal and Flint 1995), Smicridea (Smicridea) (Hydropsychidae) (Flint 1989), Tolhuaca (Glossosomatidae) (Robertson and Holzenthal 2005), and Triplectides (Leptoceridae).

As confirmed by Crisci et al. (1991), Sanmartín and Ronquist (2004), and Amorin et al. (2009) the historical biogeography of southern South America is complex. The distribution of the austral South American caddisflies support this conclusion, with a fauna pertaining strongly to a transantarctic pattern, but with perhaps older Gondwana elements, as exhibited by Sericostomatidae. The current weight of evidence described above, however, supports a more recent dispersal of this southern fauna to northern South America.

Other than in a few studies, phylogenetic hypotheses are lacking for most of the taxa reviewed above. Phylogenies of Southern Hemisphere caddisfly taxa inferred from molecular data are even fewer (e.g., Johanson and Keijsner 2008) and only one (Johanson et al. 2009) has used events-based models (e.g., Sanmartín at al. 2001, Sanmartín and Ronquist 2004) or divergence time estimates (but see Amorin et al. 2009 for a critique of molecular dating) to address historical biogeography. Given the current ease with which molecular sequence data can be obtained and with the availability of newer analytical methods (e.g., Ronquist 1997, Sanderson 2002, Zaldivar-Riverón et al. 2008), there is a wealth of hypotheses that can be tested regarding the historical biogeography of the austral caddisfly fauna once phylogenetic information is available (Santos and Amorin 2007).

## Supplementary Material

XML Treatment for Notidobiella

XML Treatment for Notidobiella parallelipipeda

XML Treatment for Notidobiella amazoniana

XML Treatment for Notidobiella brasiliana

XML Treatment for Notidobiella chacayana

XML Treatment for Notidobiella ecuadorensis

XML Treatment for Notidobiella inermis

## References

[B1] Amorin DS Santos CMD de Oliveira SS (2009) Allochronic taxa as an alternative model to explain circumantarctic disjunctions. Systematic Entomology 34:2-9

[B2] Blahnik RJ (2005) *Alterosa*, a new caddisfly genus from Brazil (Trichoptera: Philopotamidae). Zootaxa 991:3-60 10.11646/zootaxa.3609.1.2 24699570

[B3] Blahnik RJ Holzenthal RW (2004) Collection and curation of Trichoptera, with an emphasis on pinned material. Nectopsyche, Neotropical Trichoptera Newsletter 1:8-20

[B4] Blahnik RJ Holzenthal RW Prather AL (2007) The lactic acid method for clearing Trichoptera genitalia. In: Bueno-Soria J Barba-Alvarez R Armitage BJ (Eds) Proceedings of the 12th International Symposium on Trichoptera. The Caddis Press, Columbus, Ohio, 9–14

[B5] Botosaneanu L Wichard W (1983) Upper-cretaceous Siberian and Canadian amber caddisflies (Insecta: Trichoptera). Bijdragen tot de Dierkunde 53:187-217

[B6] Brundin L (1966) Transantarctic relationships and their significance, as evidenced by chironomid midges. With a monograph of the subfamilies Podonominae and Aphroteniinae and the austral Heptagyiae. Almqvist & Wiksell, Stockholm, 472pp.

[B7] Calor AR Holzenthal RW (2008) Phylogeny of Grumichellini Morse, 1981 (Trichoptera: Leptoceridae) with the description of a new genus from southeastern Peru. Aquatic Insects 30:245-259

[B8] Calor AR Holzenthal RW Amorin DS (2006) Phylogenetic analysis of *Notalina* (*Neonotalina*) Holzenthal (Trichoptera: Leptoceridae), with the description of two new species from southeastern Brazil. Zootaxa 1131:33-48

[B9] Cartwright DI (2009) *Austrotinodes* Schmid, a South and Central American caddisfly genus, newly recorded in Australia, with the description of new species (Trichoptera: Ecnomidae). Zootaxa 2142:1-19

[B10] Cranston PS Edward DD (1999) *Botrycladius* gen. n.: a new transantarctic genus of orthocladine midge (Diptera: Chironomidae). Systematic Entomology 24:305-333

[B11] Crisci JV Cigliano MM Morrone JJ Riog-Juñent S (1991) Historical biogeography of southern South America. Systematic Zoology 40:152-171

[B12] Daugeron C D’Haese CA Plant AR (2009) Phylogenetic systematics of the gondwanan *Empis macrorrhyncha* group (Diptera, Empididae, Empidinae). Systematic Entomology 34:635-648

[B13] de Moor FC (1997) An unusual caddisfly larva from South Africa, a possible member of the Triplectidinae (Trichoptera: Leptoceridae). In: Holzenthal RW Flint OS Jr. (Eds) Proceedings of the 8th International Symposium on Trichoptera. Ohio Biological Survey, Columbus, Ohio, 323–330

[B14] de Moor FC Ivanov VD (2008) Global diversity of caddisflies (Trichoptera: Insecta) in freshwater. Hydrobiologia 595:393-407

[B15] Flint OS Jr. (1967) Trichoptera collected by Prof. J. Illies in the Chilean subregion. Beiträge zur Neotropischen Fauna 5:45-68

[B16] Flint OS Jr. (1973) Studies of Neotropical caddisflies, XVI: the genus *Austrotinodes* (Trichoptera: Psychomyiidae). Proceedings of the Biological Society of Washington 86:127-142

[B17] Flint OS Jr. (1974) Studies of Neotropical caddisflies, XIX: the genus *Cailloma* (Trichoptera: Rhyacophilidae). Proceedings of the Biological Society of Washington 87:473-484

[B18] Flint OS Jr. (1976) A preliminary report of studies on Neotropical Trichoptera. In: Malicky H (Ed) Proceedings of the 1st International Symposium on Trichoptera. Dr. W. Junk, The Hague, 47–48

[B19] Flint OS Jr. (1983) Studies of Neotropical caddisflies, XXXIII: new species from austral South America (Trichoptera). Smithsonian Contributions to Zoology 377:1-100

[B20] Flint OS Jr. (1989) Studies of Neotropical caddisflies, XXXIX: the genus *Smicridea* in the Chilean subregion (Trichoptera: Hydropsychidae). Smithsonian Contributions to Zoology 472:1-45

[B21] Flint OS Jr. (1992) Studies of Neotropical caddisflies, XLIX: the taxonomy and relationships of the genus *Eosericostoma*, with descriptions of the immature stages (Trichoptera: Helicophidae). Proceedings of the Biological Society of Washington 105:494-511

[B22] Flint OS Jr. (2002) Studies on Neotropical caddisflies, LX: Three new species of the Chilean genus *Microthremma*, with a review of the genus (Trichoptera: Helicophidae). Entomological News 113:225-232

[B23] Flint OS Jr. Holzenthal RW Harris SC (1999a) Nomenclatural and systematic changes in the Neotropical caddisflies. Insecta Mundi 13:73-84

[B24] Flint OS Jr. Holzenthal RW Harris SC (1999b) Catalog of the Neotropical Caddisflies (Trichoptera). Special Publication, Ohio Biological Survey, Columbus, Ohio, 1–239 pp.

[B25] Geraci CJ Kjer KM Morse JC Blahnik RJ (2005) Phylogenetic relationships of Hydropsychidae subfamilies based on morphology and DNA sequence data. In: Tanida K Rossiter A (Eds) Proceedings of the 11th International Symposium on Trichoptera. Tokai University Press, Kanagawa, 131–136

[B26] Harris SC Armitage BJ (1997) New member of the Chilean genus *Nothotrichia* from North America (Trichoptera: Hydroptilidae). In: Holzenthal RW Flint OS Jr. (Eds) Proceedings of the 8th International Symposium on Trichoptera. Ohio Biological Survey, Columbus, Ohio, 123–128

[B27] Harris SC Flint OS Jr. (1993) Studies of Neotropical caddisflies, XLVIII; the larva of *Celaenotrichia edwardsi* Mosely, with an assessment of the genus (Trichoptera: Hydroptilidae). In: Otto C (Ed) Proceedings of the 7th International Symposium on Trichoptera. Backhuys Publishers, Leiden, 101–106

[B28] Henderson IM Ward JB (2006) Four new species of the caddis genus *Philorheithrus* (Trichoptera: Philorheithridae) from New Zealand. Records of the Canterbury Museum 20:21-33

[B29] Henderson IM Ward JB (2007) Three new species in the endemic New Zealand genus *Alloecentrella* (Trichoptera), and a re-evaluation of its family placement. Aquatic Insects 29:79-96

[B30] Holzenthal RW (1986a) The Neotropical species of *Notalina*, a southern group of long-horned caddisflies (Trichoptera: Leptoceridae). Systematic Entomology 11:61-73

[B31] Holzenthal RW (1986b) Studies in Neotropical Leptoceridae (Trichoptera), VI: immature stages of *Hudsonema flaminii* (Navas) and the evolution and historical biogeography of Hudsonemini (Triplectininae). Proceedings of the Entomological Society of Washington 88:268-279

[B32] Holzenthal RW (1986c) Studies in Neotropical Leptoceridae (Trichoptera), IV: a revision of *Brachysetodes* Schmid. Transactions of the American Entomological Society 111:407-440

[B33] Holzenthal RW Blahnik RJ Kjer KM Prather AP (2007a) An update on the phylogeny of caddisflies (Trichoptera). In: Bueno-Soria J Barba-Alvarez R Armitage B (Eds) Proceedings of the 12th International Symposium on Trichoptera. The Caddis Press, Columbus, Ohio, 143–153

[B34] Holzenthal RW Blahnik RJ Prather AL Kjer KM (2007b) Order Trichoptera Kirby, 1813 (Insecta), caddisflies. Zootaxa 1668:639-698

[B35] Holzenthal RW Flint OS Jr. (1995) Studies of Neotropical caddisflies, LI: systematics of the Neotropical caddisfly genus *Contulma* (Trichoptera: Anomalopsychidae). Smithsonian Contributions to Zoology 575:1-59

[B36] Holzenthal RW Pes AMO (2004) A new genus of long-horned caddisfly from the Amazon basin (Trichoptera: Leptoceridae: Grumichellini). Zootaxa 621:1-16

[B37] Holzenthal RW Robertson DR (2006) Four new species of *Contulma* from South America (Trichoptera: Anomalopsychidae). Zootaxa 1355:49-59

[B38] Huamantinco AA Nessimian JL (2003) A new species of *Antarctoecia* Ulmer, 1907 (Trichoptera: Limnephilidae) from southeastern Brazil. Aquatic Insects 25:225-231

[B39] Ivanov VD (1990) Structure and function of setose warts of caddisflies [in Russian]. Latvijas Entomologs 33:96-110

[B40] Johanson KA (2003a) Phylogenetic analysis of the genus *Helicopha* Mosely (Trichoptera: Helicophidae), with description of five new species from New Caledonia. Insect Systematics & Evolution 34:131-151

[B41] Johanson KA (2003b) Revision of the New Caledonian genus *Mecynostomella* (Trichoptera, Kokiriidae). Zootaxa 270:1-24

[B42] Johanson KA Espeland M (2009) Phylogeny of the Ecnomidae (Insecta: Trichoptera). Cladistics 25:1-13 10.1111/j.1096-0031.2009.00276.x 34875748

[B43] Johanson KA Keijsner M (2008) Phylogeny of the Helicophidae (Trichoptera), with emphasis on the New Caledonian species of *Helicopha*. Systematic Entomology 33:451-483

[B44] Johanson KA Kjer K Malm T (2009) Testing the monophyly of the New Zealand and Australian endemic family Conoesucidae Ross based on combined molecular and morphological data (Insecta: Trichoptera: Sericostomatoidea). Zoologica Scripta 38:563-573

[B45] Johanson KA Ward JB (2002) Re-description of the genus *Zelolessica* and of its two species (Insecta, Trichoptera, Helicophidae). Records of the Canterbury Museum 16:1-17

[B46] Li YJ Morse JC (1997) Species of the genus *Ecnomus* (Trichoptera: Ecnomidae) from the People’s Republic of China. Transactions of the American Entomological Society 123:85-134

[B47] Malm T Johanson KA (2008) Revision of the New Caledonian endemic genus *Gracilipsodes* (Trichoptera: Leptoceridae: Grumichellini). Zoological Journal of the Linnean Society 153:425-452

[B48] Morse JC (Ed)(2010) Trichoptera World Checklist. http://entweb.clemson.edu/database/trichopt/index.htm [accessed 15 March 2010]

[B49] Morse JC Holzenthal RW (1987) Higher classification of Triplectidinae (Trichoptera: Leptoceridae). In: Bournaud M Tachet H (Eds) Proceedings of the 5th International Symposium on Trichoptera. Dr. W. Junk, Dordrecht, 139–144

[B50] Neboiss A (1984) A review of taxonomic position of Australian and New Guinean species previously ascribed to *Macronema* (Trichoptera: Hydropsychidae). Royal Society of Victoria Proceedings 96:127-139

[B51] Neboiss A (1986) Atlas of Trichoptera of the SW Pacific-Australian Region, Series Entomologica 37 Dr W. Junk, Dordrecht, 286 pp.

[B52] Neboiss A (2002) A family problem with placement of *Heloccabus buccinatus* gen. nov. & sp. nov., an Australian caddisfly (Insecta: Trichoptera). Nova Supplementa Entomologica (Proceedings of the 10th International Symposium on Trichoptera) 15:195-204

[B53] Robertson DR Holzenthal RW (2005) The Neotropical caddisfly genus *Tolhuaca* (Trichoptera: Glossosomatidae). Zootaxa 1063:53-68 10.3897/zookeys.114.1405 PMC3130345 21976996

[B54] Ronquist F (1997) Dispersal-vicariance analysis: a new approach to the quantification of historical biogeography. Systematic Biology 46:195-203

[B55] Ross HH King EW (1952) Biogeographic and taxonomic studies in *Atopsyche* (Trichoptera, Rhyacophilidae). Annals of the Entomological Society of America 45:177-204

[B56] Sanderson MJ (2002) Estimating absolute rates of molecular evolution and divergence times: a penalized likelihood approach. Molecular Biology and Evolution 19:101-109 11752195 10.1093/oxfordjournals.molbev.a003974

[B57] Sanmartín I Enghoff H Ronquist F (2001) Patterns of animal dispersal, vicariance and diversification in the Holarctic. Biological Journal of the Linnean Society 73:345-390

[B58] Sanmartín I Ronquist F (2004) Southern Hemisphere biogeography inferred by event-based models: plant versus animal patterns. Systematic Biology 53:216-243 15205050 10.1080/10635150490423430

[B59] Santos CMD Amorin DS (2007) Why biogeographical hypotheses need a well supported phylogenetic framework: a conceptual evaluation. Papéis Avulsos de Zoologia 47:63-73

[B60] Schefter PW (1996) Phylogenetic relationships among subfamily groups in the Hydropsychidae (Trichoptera) with diagnoses of the Smicrideinae, new status, and the Hydropsychinae. Journal of the North American Benthological Society 15:615-633

[B61] Schmid F (1955) Contribution à la connaissance des Trichoptères néotropicaux. Mémoires de la Société Vaudoise des Sciences Naturelles 11: 117–160, plates 111–117.

[B62] Schmid F (1957) Contribution à l’étude des Trichoptères néotropicaux II. Beiträge zur Entomologie 7:379-398

[B63] Schmid F (1989) Les hydrobiosides (Trichoptera, Annulipalpia). Bulletin de l’Institute Royal des Sciences Naturelles de Belgique, Entomologie 59, Supplement: 1–154

[B64] Sukatsheva ID Jarzembowski EA (2001) Fossil caddisflies (Insecta: Trichoptera) from the Early Cretaceous of southern England II. Cretaceous Research 22:685-694

[B65] Ulmer G (1912) Die Trichopteren des Baltischen Bernsteins. Schriften der Physik.-ökon. Gesell. zu Köningsberg, Leipzig, 1–380 pp.

[B66] Wallace AR (1876) The geographical distribution of animals. With a study of the relations of living and extinct faunas as elucidating the past changes of the earth’s surface. Volume 1 Harper and Brothers Publishers, New York, 503 pp.

[B67] Ward JB Leschen RAB Smith BJ Dean JC (2004) Phylogeny of the caddisfly (Trichoptera) family Hydrobiosidae using larval and adult morphology, with the description of a new genus and species from Fiordland, New Zealand. Records of the Canterbury Museum 18:23-43

[B68] Weaver JS III, Gibon F-M Chvojka P (2008) A new genus of Philorheithridae (Trichoptera) from Madagascar. Zootaxa 1825:18-28 10.11646/zootaxa.4890.4.8 33311110

[B69] Wiggins GB (2002) Biogeography of amphipolar caddisflies in the subfamily Dicosmoecinae (Trichoptera, Limnephilidae). Mitteilungen aus dem Museum für Naturkunde in Berlin Deutsche Entomologische Zeitschrift 49:227-259

[B70] Zaldivar-Riverón A Belokobylskij SA León-Regagnon V Briceño-G R Quicke DLJ (2008) Molecular phylogeny and historical biogeography of the cosmopolitan parasitic wasp subfamily Doryctinae (Hymenoptera: Braconidae). Invertebrate Systematics 22:345-363

